# An Address to the Society of the Alumni of the Baltimore College of Dental Surgery

**Published:** 1850-07

**Authors:** James Robinson

**Affiliations:** Dentist to the Royal Free Hospital, &c., London.


THE AMERICAN

### Journal of Dental Science.

Vol. X.] JULY, 1850. [No. 4.

### ARTICLE I.

An Address *to the Society of the Alumni of the Baltimore College of Dental Surgery.* By James Robinson, D. D. S., *Dentist to the Royal Free Hospital, &c.*, London.

Mr. President and Gentlemen:

Having been honored with an invitation (from the Council of this learned and distinguished Society,) to prepare an address upon some subject bearing upon the professional pursuits of its members on the completion of this the second anniversary of the Association, I did not, I fear, duly consider the important and responsible nature of the duty I had undertaken, in accepting this flattering mark of the confidence of your Council. When I recollect the eminent literary and scientific attainments of the individual who has preceded me in this task, I feel still more forcibly my own inability to do justice to the subject, and I am reminded of the observation of one of our English popular authors, who says, "Music, artillery, the roar of cannon, and the blast of trumpets, may urge a man to a forlorn hope: ambition, one's constituents, the hell of previous failure, may prevail on us to do a more desperate thing, speak in the House of Commons, but there are some situations, such, for instance, as entering the room of a dentist when the prostration of the nervous system is absolute."

Had the ingenious author of Coningsby carried his vivid imagination one step further he might have capped the climax by instancing the position of the individual who undertakes an address to a Society of Dentists themselves!

I cannot but feel how personally truthful these observations are in the position in which I have chosen to place myself.

An address, gentlemen, under the circumstances to which I have referred, and after the eloquent and attractive oration to which on a similar occasion you listened, is, you will agree with me, a task of no ordinary difficulty. Although I cannot pretend to the ability of the one who preceded me, I yield not to him in my anxiety to promote the interests of truth and science, and the respectability of that profession of which I am but an humble member, and while he soars into the higher regions of eloquence and dives into the profounder depths of knowledge, it will afford to an humbler and less competent laborer in the same vineyard, who can merely skim the surface of the vast field of science spread before him, no small gratification to know, that while pursuing independently the same end we arrive ultimately at entirely consistent and not unfrequently identical results. It is in this spirit that I offer to the alumni my "forlorn hope," and claim at their hands, for my short comings and defects, that indulgence which they will never refuse to a professional brother and a stranger.

The subject, gentlemen, which I have chosen for observation on the present occasion, is one that I am aware is not popular among the entire followers of Dental Surgery in England, and one which I feel is likely to bring down the thunderbolts of professional wrath and indignation upon the author. It is, however, the duty of every honest and independent man who wishes well to the profession and its respectability and *status*, to shrink not from the discharge of a sacred duty. If a system be radically bad, even though it may receive the countenance and support of some respectable men, it should be at once exposed. The partial, or even general assent of a body, will not convert what is in itself erroneous or improper into a correct mode of action. No sophistry can gloss over the strong line of demarcation that separates truth from falsehood; its abettors merely become *particeps criminis*, and their conduct the more imperatively requires that the matter should be brought upon the tapis, and the system submitted to the judgment of our professional brethren in the United States.

The anomalous position which Dental Surgery occupies in England is much to be regretted. Falstaff himself never possessed a more heterogeneous or nondescript army than those who now compose the majority of dentists in England. It may be said of them, that every walk of life, every profession and trade, and every country under the sun, has contributed its quota to a staff, the qualifications of many of whom may be summed up in the remark, that, having failed in every other department, they consider themselves perfectly competent to practice as dentists. So long as this state of things endures, the respectable, educated, and really intelligent men, will be compelled to endure the disgrace of "marching through Coventry" with them, as the public have no means of distinguishing the chaff from the wheat, the quack and knavish pretender from the educated practitioner. I regret to add, that it is a well established fact, that many even of those whose position and education may be said to stand high in the estimation of their professional brethren on both sides of the Atlantic, have condescended to resort to practices that not only do the profession, but themselves, a serious and indelible injury from the pernicious consequences that almost invariably attend their adoption. I refer to the employment of mercurial compounds or amalgams for filling the cavities of carious teeth.

It would be uncharitable to suppose that the adoption of this most reprehensible practice arises from ignorance of the structure, or of the pathological or mechanical treatment of these organs. It would be equally so to suppose that they would betray themselves into the use of a material calculated to destroy these organs, as well as to affect very seriously the general constitutional health of their patients through indolence or false economy. It will probably be more correct to ascribe the prevalence of the use of mercurial amalgams in many instances to a want of the practical mechanical skill, which is so indispensable to a thorough knowledge of the profession, or from inability to master the difficulties of the manipulation, which requires a certain amount of physical exertion and expertness; or from a defect in the curriculum of study and experience, which every properly educated practitioner should undergo, and which is so important an element in the ultimate success of the dentist. But to this point I shall take another opportunity of referring.

With these preliminary remarks, I shall now proceed to introduce the subject matter of this address to the Society, viz: The system of dental education pursued in England, with a division of its component members into the various classes to which their professional standing fairly entitles them, from the properly educated dentist to the empiric who starts with an equal amount of ignorance and assurance, and who has picked up a smattering of the dental art, God only knows how, or where!

The importance and value of mechanical knowledge, in a legal point of view, has recently been exemplified in a striking manner in England, where it formed the main clue of identity in the case of murder. I allude to that of O'Connor by the Mannings, which has obtained a world-wide notoriety from the peculiar circumstances of atrocity attending it. It may be remembered, that after the victim had been despatched and buried, the body was covered with quick-lime, for the purpose of producing decomposition more rapidly, and destroying all traces of identity. A few more days, or probably hours, would have been sufficient for this purpose, had the body not been providentially discovered. At the *post mortem* examination, it was ascertained that the deceased wore a set of artificial teeth. The evidence of the surgeon who examined the body, and which contained a description of the mouth, with the peculiarity of a protruding lower law, appeared in the public papers. This attracted the attention of the dentist, who had had a similar case under treatment: the teeth were shown to him, recognized and applied to the working model, thus proving, beyond a doubt, that it was the same individual who had been murdered. The recognition and identification by the gentleman who had made and adapted the teeth, followed as a matter of course, and a most important link in the chain of evidence was thus obtained. Presuming the discovery of the body not to have taken place till after the corrosive qualities of the lime had taken effect upon the features, so that no trace of identity of the deceased could have been obtained, the conviction of the guilty parties would have been extremely problematical; the whole case would be resolved into one of mere surmise and suspicion, if the dentist had not stepped in to clear up the mystery with his mechanical knowledge, by which he was enabled to swear with positive certainty to the work which he had made and fitted to the mouth of the murdered man. Most dentists are, of course, aware, although the public are unacquainted with the fact, that there are unequivocal finishing marks by which the style of each man's workmanship is known to himself, and that a proper amount of that mechanical skill which every dentist is supposed to possess, will enable him, with unerring certainty, to recognize, even after the lapse of years, the work which he has himself made and adjusted. Without this, the culprits would, in the case to which I am referring, have probably escaped the just punishment of their crimes.

Another case of identity by means of artificial teeth, probably not so well known as that of the unfortunate O'Connor, I may here refer to. It is that of Sir William Henry Malcolm, who commanded a regiment of dragoons at the battle of Waterloo. Like many other brave fellows, he fell upon that bloody field to rise no more. During the night, notwithstanding the vigilant watch that was kept, many of the dying and the dead were stripped and plundered, and, after a lengthened search, a body, which many of his brother officers at once recognized as that of Sir William, was found, and preparations were made to give it decent burial. His orderly, an old and faithful domestic, who had been severely wounded in the engagement, stoutly maintained that it was not the body of his late master, and convinced all by opening the mouth of the dead man, and showing that a certain artificial tooth, which Sir William had taken great pains to conceal from all the world but his old servant, was wanting! Further search was made, and the body very much disfigured and scarce recognizable, but for the artificial tooth, was ultimately found and committed to its last resting place.

If we require more proof, we have only to refer to the dreadful tragedy which has more recently occurred at Boston. I allude to the murder of Dr. Parkman. In that awful case, the bones of the cranium had been calcined by throwing them into a furnace, the ashes of which were examined, and amongst them, artificial mineral teeth were found mounted upon gold, which could not be destroyed. Enquiry was made amongst the dentists, and Dr. Keep, a celebrated dentist of the place, instantly identified the work, placed them upon his working model, and at once supplied an important link of evidence, he having made the teeth a few weeks previously.

These instances, out of many that might be cited, are interesting, as showing how important and intimate a connection there exists between a proper knowledge of the practical and mechanical department of the dental art, and its application as an auxiliary of medical jurisprudence, in which it can be rendered subservient to furthering the ends of justice. Such cases place the value of the dental art in our social state in a new point of view, and it will naturally recur to the minds of all who hear these observations, how could a merely theoretical dentist identify pieces of artificial work which he neither knows how to make nor to adapt to the mouth, or how could he be supposed capable of identifying a filling of gold or any of those minute characteristics, in the shape and arrangement of the teeth, which none but a practical and intelligent dentist can be thoroughly acquainted with. The case of the Mannings occurred recently; who can say how soon or under what circumstances another appalling tragedy of a similar nature, a suicide, or other violent case of death may not occur, in which a knowledge of mechanical dentistry may not be invoked to establish identity. And it should be borne in mind, that it is not alone by means of artificial teeth that this may be accomplished: the mode of plugging, filing, finishing, and the general form and character of the teeth themselves will all suggest to the close and attentive observer, points of recognizance by which identity may be arrived at.

I have already pointed out, in my work on the Teeth, the practical importance of a knowledge of dentonomy, or the physiognomonical harmony and keeping that prevail in the teeth as in the other parts of the human body. Recent observation has confirmed, in every particular, the suggestions I there offered to the consideration of the profession, and which I shall not now repeat; but all who have taken the trouble to investigate and examine the subject will agree with me as to the distinct peculiarities that characterize the mouth of each individual, and which are so well defined that a set of artificial teeth will only fit the mouth for which they were designed. Indeed, there is as great, if not greater variety in the mouths and teeth of individuals, as in their faces and characters.

But to return from this digression, which the interesting nature of the subject led me into, I shall now, before proceeding to consider the system of dental education in England, briefly advert to a few of the early authors who have written upon this subject, and who may be said to have been the historians of the theoretical part, and the pioneers to the accurate knowledge we have now obtained of the anatomy, physiology, and scientific treatment of the various diseases of these organs.

It will be remembered that Burdmore was the first writer in England, so far as we are aware, who drew attention to the subject, or systematized the practice of Dental Surgery, in the year 1770. It was he who first drew attention to "the disorders and deformities of the teeth and gums;" and although his notions were somewhat crude and at variance with our present knowledge, his writings at this early period exhibit a somewhat extensive practical acquaintance with the subject upon which he wrote. Subsequent writers have not, I think, given Burdmore that fair share of credit and praise to which he is entitled, if we for a moment consider the untrodden path of science into which he was the first to adventure. Burdmore found the practice of Dental Surgery, such as it then was, in the hands of barber surgeons, shoemakers and mountebanks, and their knowledge of the subject as narrow and circumscribed as was their practice, it being limited to the mere extraction of teeth.

It was in this primitive state that Burdmore found the dental profession; he raised it from obscurity, and made it of sufficient importance to attract the attention of the celebrated Hunter, who wrote in 1771. Although Hunter did turn his attention to the subject, nevertheless Dental Surgery advanced but slowly, and all the theoretical knowledge that existed upon the subject up to that time, was derived from Hunter's works.

In 1803 the work of Fox appeared, which not only treated the teeth medicinally and surgically, but also embraced a portion of the mechanical treatment; and the novel and interesting field of inquiry which it opened up, naturally attracted the observation of the scientific and curious. For many years subsequently the art appears to have remained in abeyance, medical pupils being too much engrossed with their studies upon General Anatomy and Operative Surgery to pay any serious or continuous attention to this part of the human frame. In fact, specialists who devoted themselves exclusively to the knowledge and study of one portion of the body, were looked upon, even at this period, with something like suspicion and distrust, and the treatment of the teeth by medical men was confined to mere extraction.

Although a few abstract treatises followed these standard works, it was not until the appearance of the scientific publication of Bell, in the year 1829, upon the Anatomy, Physiology, and Pathology of the Teeth, that Dental Surgery may be said to have engaged any particular attention, or to be studied as an important collateral branch of Medicine and Surgery. It was only then that the teeth were first pointed out as an integral part of the human economy, and as such, entitled to form an important element of consideration in the treatment of nervous diseases of the cranium. Works of a similar theoretical character had appeared upon the Continent, but no work so comprehensive and perfect as that of Bell had yet been produced. The author, a man of profound physiological and pathological observation and acquirements in the departments of human and comparative Anatomy, could not have failed to produce a work worthy of his correct views, his eminent position and his great talents. Although some of his theories have within the last few years been questioned by modern writers; yet, as a whole, the talent and research it displays are universally admitted, while, as a sound, theoretical work, it has never been surpassed.

It may, perhaps, be scarce necessary to mention, that some valuable works supporting the views of Bell subsequently appeared, and numerous smaller compilations and contributions to this department of science, in the shape of pamphlets, of which it is unnecessary to mention more than the titles. The year 1830 was prolific in these ephemeral publications, varying in price and quality of matter. We had "The Mother's Guide," "The Infant's Friend," "Five Minutes' Advice," and a host of others of similar kind. It cannot be denied that works of a popular character have a great tendency to do good, if they be the productions of men who know how to convey the requisite amount of information in a popular and intelligible form, and if the principles laid down be sound and practicable. The attention of the public is thereby attracted to the subject; just and intelligent views are formed of the value and importance of organs, which are too frequently neglected till disease has done its work; and the character of the profession is elevated in the eyes of the public by a more general diffusion of the sound and scientific knowledge and skill which are requisite for its judicious exercise. Unfortunately, in England, the absurdities propounded by egotists neutralize the good effects that might otherwise be produced; and in the race for honors and practice, the really deserving but diffident man is too often jostled out of the path by the more noisy and boastful claimants upon public patronage.

Subsequently to this period, we have had a number of *theoretical* works emanating from men, whose acquirements were well calculated to increase the importance of Dental science by their researches in physiology, but hitherto not a single work of a thoroughly *practical* character. The field was occupied by speculations, theories, professional squabbling, and the publication of abstract papers, while those best calculated to communicate valuable information to the profession, hung back from an unwillingness to encounter the rough handling and idle bantering which several theoretical practitioners had undergone, and kept the knowledge which they had acquired, like the *modus operandi* in their workshops, a secret from the rest of the world.

It is not surprising under these circumstances, that the practical department of Dental Science, should remain stationary while the theoretical portion progressed. No work calculated to meet the requirements of the profession had yet appeared, and the student who wished to acquire a knowledge of the whole system knew not where to turn—the practical and mechanical, by far the most important portions of the art, were shrouded in mystery, and their details left to chance or experience. Students who had become acquainted with the mechanical, and wished to extend their knowledge of the theoretical and practical portions, after the usual routine of study at the hospital, or elsewhere, were left in helpless ignorance. Young men, it is true, attended lectures on Dental Surgery, but beyond anatomy, physiology and pathology, no information was acquired. There was no practical illustration, (excepting the abduction of teeth) no demonstrations either upon the living or the dead subject, to guide the young practitioner in his future career. From the period of his entering the Dental Lecture-room of the Anatomical Theatre, in which those lectures are delivered subsequently to the lectures on General Anatomy, the pupil who enters solely for lectures upon Dental Surgery, and they are very few, learns nothing, or almost nothing, of a practical nature. While those who are attending the hospital for a general medical education seldom trouble the dental lecturer with their attendance, being too much engrossed with those more important studies (upon which they know they will have to undergo an examination, previously to obtaining their diploma,) to pay any attention to the dental organs. In fact, it was, and I believe still is, considered *derogatory* to a student to pay particular attention to any exclusive department that was likely to lead him to specialism. Even the lecturers themselves, in their discourses, allude in the most cursory manner to the mechanical branch as if, being handicraft, it were of no importance; although all who know anything of Dental Surgery must be aware, that it cannot be advantageously or efficiently practised without mechanical knowledge.

I have elsewhere pointed out the intimate relation that exists between Dentistry and Mechanics. I should recommend to the attentive consideration of the student or youthful practitioner the few remarks I have ventured to offer, in the work to which I allude, upon the subject of dental education, contenting myself here with repeating the observation I therein made, that "the dental art, in its perfection, is the very beau ideal of pure mechanical surgery." From the facts I have just detailed, and the generally received opinion that a knowledge of Dental Mechanism is not absolutely necessary, it is not surprising that ignorance with regard to the most essential elements of Dental Science should prevail in the medical profession. Unfortunately for our advancement as a body, an injurious impression is abroad that any person is fit to practice as a dentist; his responsibility is so slight, his calling so insignificant, and his knowledge so limited and so easily acquired. In many instances, a personal knowledge of parties who have turned dentists, whatever infinitisemal amount of practice they may obtain, is brought forward in support of these observations. A biographical living sketch-book of the dental practitioners of England would form a curious and amusing melange, and would show the incongruous materials of which the craft is composed. We should have blacking-merchants, Jew clothesmen, Jew bill-discounters, policemen, horse-dealers, milkmen, broken-down doctors and apothecaries, rejected medical pupils *cum multus alus.* The more ignorant and unscrupulous among them adopt some high-sounding French or jaw-breaking German name, (emblematical, in all probability, of their future operations,) and shroud their grub-like origin in the butterfly hues of a flaunting equipage and flaring liveries to dazzle the eyes while they empty the pockets of their dupes. Such is the varied character of the dental profession in England, which boasts of her wealth, her civilization, and intelligence, while she bows down before the meretricious shrine of quackery and humbug.

It must, however, be distinctly understood, that in making these observations I have no intention, as I certainly can have no wish, to cast the remotest reflection upon those who, having pursued avocations of a similar character, have, by natural talents, perseverance and education, raised themselves to eminence in a profession for which their previous education had fitted them. My observations apply to those who have become dental practitioners in a month, and, in many instances, in a few hours, the metamorphosis resembling the changes in our Christmas pantomimes, *exit* Mr. Mordecai from Hounsditch, and *enter* Monsieur de St. Aulaire in the aristocratic precincts of Mayfair. These men avail themselves of the proffered services of advertising professors, who manufacture dentists in England at 5*s.* per lesson, and at the end of a month or two let loose their hopeful pupils upon society, with the assurance, by no means unfounded, that they are as competent as themselves to practice as dentists. What is so easily acquired may be as readily imparted, and accordingly these gentlemen in their turn become the teachers of pupils, give instructions upon the same principles and at their full value, namely, very reduced prices; the consideration being, in some instances, a few bottles of gin or whiskey, or some commodity equally suited to the tastes of the "profession." Indeed, a person in

England desirous of following Dental Surgery, has only to pay a few pounds, learn to take an impression in wax, a cast, plaster, fill carious teeth with amalgams, provide himself with a few instruments, the names and uses of which it is not absolutely necessary he should be acquainted with, a chair, basin, &c., stick his name on a brass plate, reserving for the operating room a supply of the same commodity, use hard names, and exhibit to the unfortunate simpletons who call, the identical instrument which had been applied to the jaws of the Emperor of China or Queen Victoria, hinting that as a great favor, which must not be mentioned to their most intimate friends, it shall be used upon themselves on that particular occasion. The bait will, upon the first performance, invariably take, and the simple dupe pays handsomely for the regal honor.

It is gratifying to find that since the foundation and progressive advancement of the American Society of Dental Surgeons, and the publication of its transactions, great improvements have taken place in the practice of the best class of practitioners. The establishment of a society exclusively devoted to the promotion of the interests of Dental Science and Dental Literature, the publication of its proceedings, inventions and discoveries, and the combination of the most learned and respectable of the profession of an entire nation, have stamped the reputation and secured the influence and authority of the Society. It has thrown open the resources of our art, diffused among the profession the results of its intelligence, its research and its experience, and placed Dental Surgery upon the broad and comprehensive basis of scientific truth. Its value and importance will henceforth be measured, not by the depreciating estimate of those unworthy members who are dentists only in name, but by the high intellectual standard of those who in the formation of the Society, have proved themselves at once the ornaments and the safeguard of our professional character and reputation, and the guarantees that our merits and our services will be duly appreciated and acknowledged by the public.

It must be confessed that our science was not generally understood or its resources made apparent and available until within the last fifteen years. There were a few, who, by great natural talents and perseverance, excelled either as theorists or mechanists, and who obtained the just reward to which their reputation and ability entitled them. But no defined or correct system of practice had been drawn, no curriculum of study had been prescribed either in dental operations, or in mechanics. Each practitioner plugged teeth, took his models, or manufactured his sets after his own fashion, conducting the whole of his proceedings with the most profound mystery and secrecy. Each man regarded his neighbor as a professional rival, the great aim being to keep his mode of practice to himself, and not unfrequently the meanest and most knavish tricks were resorted to, to revenge the success of a rival. Instances were not unfrequent in which the enterprising or curious practitioner who sought the privilege of examining the arrangement, details or *modus operandi* of the workshop or surgery, has been subjected to abuse and rough usage—the ordinary courtesies of professional life being entirely abrogated in dental practice.

Since the subject of Dental Surgery has been thrown open, and the portals of the science widened for the admission of all who seek information, a more liberal spirit has diffused itself among the more educated and respectable practitioners. A greater ambition to pursue investigations upon known and intelligible principles, and a greater readiness to communicate the results of their study and experience exist. To this improved state of things, there are a few unworthy exceptions among men, who, although professing education and station, demean themselves by resorting to low cunning and unprofessional practices, and do not hesitate to claim for themselves all improvements and discoveries in every department of the dental art. It was a frequent practice some years since, after the formation of the American Society, and the issue of its publications, for tricksters to fasten upon some original matter or a point of dental practice, culled from the Journal of the Association, and forwarding it to some of the medical publications of this country, foist it upon the world as their own. The American Journal having at that time but a limited circulation in England, and being little known among the profession, the plagiarist enjoyed for a time the fruit of his dishonesty until the trick was discovered—when the lion's skin fell from his shoulders, and his long ears and his uncouth braying revealed his inordinate vanity and his asinine stupidity.

The leading class of dental practitioners in England, to which we are now about to refer, includes among its members many men, who, possessed of a sound and liberal education, a well regulated mind, and a natural taste for the profession, have, by their energy and perseverance, attained a deservedly high position both as theorists and practical men. Their knowledge of the profession may be said to have been acquired from actual experience and observation—possessed of a general knowledge, and being well read in the theoretical works of the day, they felt the necessity of laboring diligently in order to obtain the necessary amount of practical information, as they soon become convinced that in order to pursue the profession with any chance of ultimate success, such knowledge was indispensable to save them from blundering at every step of their progress. There was no practical work of any value published at the time they entered upon their professional career, from which the slightest information could be gleaned—the text books from which they studied were chiefly upon the subjects of Anatomy, Physiology and Pathology—all very well in their way, but like the receipt for making plum-pudding, liable to error without practical experience in cookery. The story may not be so familiar on your side of the Atlantic as it is in England, that when the dashing Marquis of N——y was ambassador at Paris, a few years since, Louis Philippe having invited him to dinner on Christmas day, was anxious to pay a compliment to the national taste of his distinguished guest by treating him to a splendid plum-pudding *à l'anglaise.* The most approved receipt was procured from London, and confided with many solemn injunctions to His Majesty's *Chef de cuisine.* All the necessary ingredients were prepared and mixed *secundem artem,* and when the eventful moment arrived, His Majesty gave the signal to serve up the English plum-pudding. A huge silver tureen was placed on the royal table, evidently amazed at the strange company in which it found itself towering over the *entremets soufflets* and delicate *petits-plats,* that graced the banquet, and a soup plate filled with some savory mess was placed before the noble Marquis, with many pressing entreaties to partake of his national fare. It is almost needless to finish the story—the cook had forgotten the *pudding-cloth,* and had boiled the ingredients in the water, and to this day the Marquis tells with much gusto, the astonishment, annoyance and regret of his Royal Host at the failure of the kindly meant compliment. When a royal cook, and that cook a French one, so signally failed by relying merely upon his theoretical skill, our young students and practitioners may well pause and ponder upon the serious responsibility they incur when they venture to apply their mere theoretical knowledge to practical purposes, and this is the moral I wish to draw from my anecdote.

In England, it may be said that with energy and perseverance the student in practical and mechanical Dental Surgery commenced his studies, with assiduity and diligence accomplished his task and soon discovered the value of the information he had thus obtained in the practice of his art, rendering its details and the various operations required of him comparatively easy. Although it must not be supposed that the perfection of the present day had as yet been attained, it will be admitted by those who know the difficulties they had to contend with in procuring the requisite information that great credit is due to them for the high position to which they carried this important branch of the art. Little of the egotism and absurdity that marked the career of their predecessors now remains, and few among them are silly enough to parade pseudo-royal instruments to endeavor to elevate themselves by depreciating the acquirements or decrying the merits of their brethren or entertain the ridiculous notion that they are beyond the reach of improvement. On the contrary, I am happy to say that most of them continue to watch and study the improvements of the day with the same marked interest and assiduity as at the commencement of their career, and have shewn themselves the true students of dental philosophy, the lovers of that wisdom and that information that is to fit them for their occupation, the searchers after and the investigators of scientific truths.

It will be inferred from what I have stated that much has been effected in England within the last few years, but I regret to add that much more yet remains to be accomplished before Dental Surgery can occupy its fair and legitimate position as an important department of medical science. A dark cloud overhangs the profession, and involves even the most eminent and distinguished in its shadow. High as their social rank or professional attainments may be, they are open to the imputation of having made no effort collectively or individually to imitate the noble example set them by the dentists of the United States, and form an association or society of the leading men of their body, and by united exertion rescue the profession from its present questionable and somewhat equivocal position. Every other department of medicine and surgery, nay every profession with any claim to public consideration, has its tribunal, before which the qualifications and respectability of all seeking admission, are tested and scrutinized—the dental art remains as yet the sole and humiliating exception; the leading men are content with the reputation and the position they have acquired, under the pretext that their numbers are too few to be able to affect much good, but unless that few combine for the benefit of the profession at large, it can never be effectually rescued from the cloud of quackery and imposition which have hitherto obscured it, and from which it is the duty of the leading men to free it. Instead of using their legitimate influence, and raising their voices to purge it of pretenders, they by their culpable silence give an indirect sanction and covertly uphold the practices that have lowered it in public estimation. I would willingly have passed over these observations that taints and tarnishes the whole body to some extent, but much as I owe to my country and to my profession, I owe still more to the interests of truth and of justice, and I cannot conceal or gloss over such glaring imperfections. I have only to express a hope that we may shortly see the dawn of a better day and a more satisfactory state of things for Dental Surgery in England.

Many of the accomplished men to whom I have alluded, are fast disappearing from the busy stage of life—a few still remain as "bright particular stars" for the guidance and enlightenment of their younger brethren, pursuing their honorable course with integrity and intelligence, eschewing all discreditable trickery and meanness, lending a kind and a helping hand to those yet climbing the steep and rugged paths of professional distinction, and long may they remain with us honored and respected, to shed a lustre and a dignity upon the art of which they are the fathers and the protectors.

Before closing the subject of Dental Surgery in England, I shall say a few words upon the education of pupils, in order to show what are the future hopes and prospects of the profession. A pupil, say fifteen or sixteen years of age, desirous of learning his art

*thoroughly,* seeks out a respectable practitioner in full practice, who invariably requires an apprenticeship of five years—the first three or four being devoted solely to learning mechanical dentistry, at the end of which he attends the practice of a public institution, and hears lectures upon Medicine and Surgery, devoting his spare time to the continued practice of mechanism at the work bench. After a certain period, determined by his dexterity and acquirements, he commences the operative part at the hospital, and this he continues with close study till the termination of his apprenticeship. In some instances, the practitioner introduces him to his private surgery, by which means, confidence, address and practical skill are acquired, by either witnessing operations, or having cases entrusted to his management, under the eye of the professor. When the articles expire, the student having entered for the lectures, can, if he please, present himself for examination at the college, if he thinks that the diploma will add to his position as a dentist. Few pupils educated in this way and under such favorable auspices, think the diploma worth the trouble and expense, for reasons I shall mention, when speaking of another class of practitioners.

Pursuing my enquiry, I now come to another description of dentists. I wish I could add a better, or even so good as the preceding one. Like the showman who advertised in a country town the exhibition of an extraordinary animal, called the Worser, "never before exhibited," and when he had collected a numerous audience of gaping clod-poles, and produced two dogs, one an indifferent specimen of the canine race, and the other the most miserable starved and mangy specimen that could be procured, he coolly observed "now ladies and gentlemen, I call that ere dog a tolerable good dog, and that ere tother one the worser," and then decamped with the coppers; so I am compelled to say of the lot now under consideration, that "this here tother lot is the worser!" It includes many disreputable specimens of gentry, who have tried their hands (I fear they never had any heads) at every description of trade not likely to be serviceable to a dentist. Some emerge from behind a linen-draper's or book-seller's counter, or terminate in it their wandering career, as commercial travellers. Turned over or turned off apprentices, or assistants, from either incapacity or disinclination to follow the more humble and suitable avocation, and medical students intended for a purely medical profession, who lack brains to master even the initiatory knowledge.

Numbers again, who attend medical or surgical lectures, having followed no previous occupation, and who from want of attention or habits of industry, have little chance of obtaining their diploma—the period of examination arrives, in many cases a sufficient number of lectures have been attended to ensure the necessary certificate of attendance. Notice of appearance for examination is registered, the aid of those valuable functionaries, Messrs. Grinder & Crammer, is called in to rub up and polish the neophyte in his perilous passage across the *pons asinorum* of Surgery. The time required for this preparatory training depends of course upon the assiduity of the candidate or the studies he may have commenced at the hospital. Some, doubtless, pass with honor and credit, while others are like an objectionable bill in the House of Commons, is ordered to be read that day six months. Few of this latter class have the courage to make the attempt again. Among the vast number of medical graduates turned out annually by the colleges, some succeed as medical practitioners, but many fail; those who are rejected without obtaining a diploma, and without a legitimate profession or the means of obtaining an honest livelihood, turn specialists, and from this class our ranks are largely recruited, dentistry being the department usually selected to exercise their talents.

The adage that any fool will do for a parson or a physician, may be applied with still greater force and truth to the profession of a dentist. We have government interference in all matters relating to the well being of society at large; we have sanitary regulations for the purification of the air we breathe; the water we drink, and the houses we inhabit—the butcher, the baker, the lawyer and the brewer, are all interdicted the use of deleterious ingredients, under certain pains and penalties, but the human body is in England left to the tender mercies of every humbug and charlatan to exercise his noxious craft upon, and we cannot wonder then that the teeth being an integral, an important portion of the body, should be equally overlooked and neglected. This will, I fear, be the case so long as the profession neglects its own interests—there will doubtless be some difficulty in cleansing the Augean stable—the vices and the errors of a system which has taken such deep root in our society, cannot be eradicated in a day, but if once a legislative enactment were procured requiring a certain standard of education, defining a certain curriculum of study, and rendering imperative a certain preliminary examination before a license to practice could be obtained, the profession would soon rid itself of the odium that attaches to it, and take its proper and legitimate position among its kindred departments of Medicine and Surgery.

Apart even from the number of charlatans, there are a large class, who, educated as medical men, have acquired their whole stock of knowledge from occasional attendance at the dental theoretical lectures, or perhaps may have a friend a dentist, and learn by words, or an attendance resulting more from curiosity than any thing else. Unless originally intended to practice as a dentist, it cannot be supposed that the medical pupil will devote any considerable portion of his time to the study of a subject which he knows he will not be examined upon, and which he will not be required to apply practically beyond the extraction of teeth. He has no time in the round of study more immediately connected with Surgery and Medicine, to learn any thing of dentistry beyond a mere rudimental knowledge—he may know how to tie the carotid artery, amputate the leg, or give you a dissertation upon disease of the ancle joints, or the sinovial membrane, but he knows little or nothing of the diseases or practical treatment of the teeth—his information being on a par with that of the gentleman, who being asked if he could speak German, said "no! but he had a brother who played very well upon the German flute."

In England, the dental art has always been looked upon as an easy and lucrative profession—all the knowledge supposed to be requisite, being merely that of extracting teeth, filling the cavities with cement, taking impressions, filing, scraping and ordering lotions and powders—the handicraft of the profession, making teeth to a model, can be done, they say, by any mechanic, and they are readily adapted to the mouth. These men appear to have little idea of the extensive knowledge and practice of the mechanics of the art, even for what is termed the simple process of plugging a tooth with gold, that is requisite to accomplish it successfully and creditably, or in order to adapt artificial teeth to the mouth, even providing they are accurately made to the model, to say nothing of the practical skill required to regulate and adjust teeth by mechanical means.

With this limited information as to the necessary requirements of the dental practitioner, which characterises a large portion of the English dentists, it is obvious that the profession cannot be followed out in the judgment or skill, and that its results cannot be very beneficial to the patient, whatever it may be to the professor. Cements, compounds and melted sulphur, are the fillings generally employed—the mechanism displayed is merely that of removing from a model pieces of artificial work, and placing them in the mouth, the proper adaptation necessary to comfort and stability being in most cases beyond the comprehension, as it is beyond the knowledge of the practitioner; he can no more carry out the arrangement in its perfection, than the itinerant wooden clock-makers, immortalized by your humorous and able countryman, "Sam Slick," can regulate an admiralty chronometer. Men with such limited acquirements and superficial knowledge of the ground-work of their art, no matter what position they happen to fill, whether they obtain practice by a lucky chance, or whether by payment of a large premium, they endeavor to secure the business and connexion of some deceased practitioner of eminence, are sure to find their true level at last. They rarely succeed in retaining the confidence of the old patients, or obtaining the confidence of new ones. They continue for some years to practice upon the strength and reputation of their predecessors, but eventually the counterfeit is found to be false and hollow, and they are compelled to retire, not with a fortune, nor with the good wishes of their patients, but with the chagrin and mortification of disappointed ambition and reduced means.

I have been induced to dwell upon the character, the pretensions and the career of this class at greater length than their importance warrants, in order to exhibit them as a warning to the rising generation of dentists and the young aspirants for professional distinction. I trust the picture I have sketched may have the sanitary effect of showing them the inutility of relying upon either the patronage of others or their own pecuniary resources. One or other of these auxiliaries may, for a time, give them an ephemeral position, but if they neglect the more legitimate and essential means of securing it by acquiring a thorough knowledge of the practical and mechanical branches of their profession, they will inevitably descend from their improperly-acquired elevation more rapidly than they attained it, while the more steady and persevering practitioner will, perhaps, more slowly but more certainly attain the status and practice to which he is fairly entitled. As I have counselled them to eschew all sudden and rapid, because illegitimate, modes of advancement, so I would earnestly entreat of them to shun the conceit and vanity of those half-educated men who, by swagger and braggadocio, by fawning and subserviency to the great and unworthy—by depreciation of the merits of all whom they look upon as rivals, endeavor to make up for their own ignorance, and to obtain practice by arts unworthy the lowest grades of the profession.

Another class who by courtesy are in England called "dental practitioners," and who are as numerous as they are ingenious, brings us to the lowest grade, in which the absolute dearth of all professional knowledge is poorly compensated for by a gentlemanly garb, with an extra allowance of jewelry and ornaments. Setting up in handsome reception rooms, with a footman to attend and advertise, "the dodge" enables two humbugs to drive a considerable trade, the servant of to-day being the operator of to-morrow, and *vice versa*, like Archer and Aimwell in the Beaux Stratagem, "I master at Lichfield and you master at Coventry." When the duties of the day are over, the worthy partners in the firm retire below, the rooms being only hired by the week during business hours, with the understanding that the kitchen is to be their sphere for the remainder of the day. To call upon one of these professors, either Jew or Gentile, after reception hours, is one of the curiosities of professional life that few have fathomed. The practitioner is found transformed into a species of light porter, or it may be that he has an indirect interest in the returns of some milk-walk, a second-hand clothes-shop, or endeavors to eke out the business of the day by persuading you to strike a bargain in teeth, or exhibit some prime "Havannas," steel pens, a horse, a Rembrandt or a Murillo. Nothing comes amiss to these gentry, who, if they have no other merit, must be set down, at least, as very industrious and very persevering.

The outline I have drawn of this class will probably appear, in the eyes of your Society, as savoring of exaggeration and caricature. I can assure you, however, that most of my portraits are drawn from life, and that it would require a Cruikshank or a Madows to delineate their peculiarities and bring their salient points prominently before you, or the graphic powers of a Jerrold or Lemon to finish biographical sketches which would do justice to the strange and incongruous mass who represent so large a portion of the profession in England. Probably the only redeeming quality they possess, is that of advertising their own merits, when others, little removed from them in scientific acquirements and qualifications, resort to more covert and underhand means to effect the same object, their sensitive delicacy shrinking from the degradation of a direct appeal. And certainly, of the two classes we should prefer Sir John Falstaff's ragged regiment of scarecrows who openly profess wonders, both mechanical and surgical, at the cheapest rate, so that those who consult them do so for a specific object, that of economy, and to obtain the largest amount of work for the smallest sum. To the large number of persons who prefer quantity to quality, the cheap advertising dentists appeal not in vain.

Enough has probably been stated to give the members of your Society some idea of the anomalous and unsatisfactory state in which the dental art stands in England; that it ought speedily to be put an end to must be sufficiently obvious from the fact, that the lowest, the most ignorant, and the most illiterate of the class to which I have referred, are nominally members of the same profession, and occupy publicly precisely the same position in England, Ireland and Scotland as any member of your Society, however eminent he might be, would fill, if he became a practitioner in the British Isles. Many details connected with this subject press themselves upon my attention, but the length to which this address has already extended warns me that I must not, perhaps, further trespass upon your indulgence and patience. My zeal in what I feel to be a good cause has led me, in all probability, beyond the limits prescribed by the rules of your Society, but enthusiasm has never been fettered by ordinary laws, and feeling deeply interested, as I do, in the well-being and advancement and respectability of a profession of which I am an humble member, I shall spare no exertions, I shall hesitate at no sacrifices, that may aid in rescuing it from the odium that attaches to it in England, and placing it in the high and honorable position which it occupies in the United States.

The conclusions I draw from the foregoing remarks may be summed up in a single sentence. I believe that Dental Science has not advanced *so generally* in England as in the United States, although this home truth may not be very palateable to the profession generally, but I make the assertion advisedly and without in the remotest degree reflecting upon the many able and scientific men in England who may be said to represent the very quintessence of dental knowledge and mechanical skill in their own country. I feel satisfied that until the barriers of professional jealousy and private interest are broken through, until a kindly and more liberal feeling is established among the leading members of the profession, until unanimity of purpose and honesty of intention prevail, until a college of Dental

Science is established and competent examiners appointed to test the qualifications of all aspirants, and ascertain that their claims to practice as dentists are founded upon a sound and solid basis, Dental Surgery can never hold its proper position in England, or assume its legitimate place by the side of its parent science, Medicine.

Mr. President and Gentlemen: Permit me, in conclusion, to tender the respectful tribute of my admiration to your enlightened body for the varied and important services it has rendered to the cause of science generally, and to that department of it with which we are all more immediately connected, in particular. By your conduct you have set a noble example to other countries, and as you have achieved your own position and asserted the dignity of your calling by your own unaided exertions, I have no fears for the future prospects or the future destinies of Dental Surgery in America, or that the heads and hands that have raised the goodly superstructure will not be at all times willing and able to defend it from the attacks of pretended friends or avowed enemies. Confiding this paper to the hands of my esteemed friend, Dr. P. H. Austin, who has kindly undertaken to deliver it, I now severally and collectively wish you all the happiness and prosperity to which your talents justly entitle you. I respectfully bid you—FAREWELL!

